# 

**Published:** 1873-03

**Authors:** 


VOL. III.] MARCH, 1873. [No. 3.

### THE SOUTHERN MEDICAL RECORD:

A MONTHLY JOURNAL OF

### PRACTICAL MEDICINE.

EDITED BY

T. S. POWELL, M.D.,

AND

W. T. GOLDSMITH, M.D.

*"QUICQUID PRÆCIPIES ESTO BREVIS."*

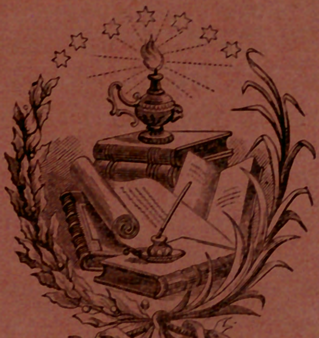

ATLANTA, GEORGIA:
PLANTATION PUBLISHING COMPANY'S PRESS.
1873.

TERMS, $2 50 PER ANNUM, IN ADVANCE. SINGLE NUMBER, 25 CENTS.

